# Sugammadex in systemic mastocytosis: Case report and a systematic review of literature

**DOI:** 10.1007/s00101-021-01069-5

**Published:** 2021-11-09

**Authors:** A. Becerra-Bolaños, V. Muiño-Palomar, S. Cabrera-Doreste, A. Rodríguez-Pérez

**Affiliations:** 1 Department of Anesthesiology, Hospital Universitario de Gran Canaria Doctor Negrín, C/Barranco de la Ballena S/N, 35010 Las Palmas de Gran Canaria, Spain; 2 Department of Medical and Surgical Sciences, University of Las Palmas de Gran Canaria, Las Palmas de Gran Canaria, Spain

**Keywords:** General anesthesia, Mast cells, Neuromuscular agents, Tryptases, Perioperative monitoring, Anästhesie, Mastzellen, Neuromuskuläre Wirkstoffe, Tryptase, Perioperative Überwachung

## Abstract

Perioperative management in patients suffering from systemic mastocytosis is challenging. Most recommendations regarding anesthetic management in these patients are based on clinical reports, and there are controversies about the use of rocuronium and sugammadex. We present a case report of a patient with systemic mastocytosis who was given sugammadex for rocuronium reversal. Tryptase levels were monitored during the first postoperative 24 h, without evidence of elevation. We also performed a systematic review to provide an overview of current evidence regarding the safety of using sugammadex in patients suffering from systemic mastocytosis. The search strategy included PubMed and Google Scholar. All studies published up to and including January 2021 concerning anesthetic management in systemic mastocytosis were included. Of the 122 articles located, 9 articles were included: 2 reviews and 7 case reports. Data from reviewed studies confirm that sugammadex can safely be administered in patients suffering from systemic mastocytosis.

## Abstract (de)

Die perioperative Behandlung von Patienten mit systemischer Mastozytose ist eine Herausforderung. Die meisten Empfehlungen zur Anästhesiebehandlung von diesen Patienten basieren auf klinischen Berichten und es gibt Kontroversen über die Anwendung von Rocuronium und Sugammadex. Wir präsentieren einen Fallbericht über eine Patientin mit systemischer Mastozytose, der Sugammadex zur Rocuronium-Umkehr verabreicht wurde. Die Tryptasespiegel wurden während der ersten postoperativen 24 h überwacht, wobei keine Erhöhung dieser Tryptasespiegel beobachtet wurde. Es wurde auch ein systematisches Review durchgeführt, um einen Überblick über die aktuelle Evidenz zur Sicherheit der Anwendung von Sugammadex bei diesen Patienten zu geben. Die Suchstrategie umfasste PubMed und Google Scholar. Alle Studien zur Anästhesiebehandlung bei systemischer Mastozytose bis einschließlich Januar 2021 wurden eingeschlossen. Von den 122 gefundenen Artikeln wurden 9 Artikel aufgenommen: 2 Reviews und 7 Fallberichte. Die Daten aus überprüften Studien bestätigen, dass Sugammadex bei Patienten mit systemischer Mastozytose sicher verabreicht werden kann.

## Introduction

Systemic mastocytosis is a rare disorder characterized by abnormal proliferation of mast cells in tissues and organs. During the perioperative period, the presence of stimuli is frequent. Stressors such as tissue manipulation, alteration of body temperature, pain, or administration of anesthetic/analgesic drugs can provoke mast cell degranulation. Releasing mediators such as histamine, tryptase, prostaglandin D2 and heparin can produce severe systemic manifestations [1, 2]. Therefore, the anesthetic management of these patients is dangerous and challenging; however, most of the anesthetic recommendations are based on individual clinical cases due to its low prevalence.

One of the most current controversies in the field of anesthesia for patients with mastocytosis is the use of rocuronium and sugammadex [3]. Both rocuronium and sugammadex may be one of the medications most frequently involved in perioperative anaphylaxis, and they are potential triggers of mast cell degranulation. Thus, among patients investigated for suspected anaphylaxis in a retrospective study performed in the Japanese Gunma University Hospital region, this was confirmed in 21.7% for rocuronium and in 28.3% for sugammadex [4]. Besides, rocuronium has been shown to cause mast cell degranulation by activation of a specific mast cell receptor [5], but the underlying mechanisms by which these drugs cause anaphylactic/anaphylactoid reactions in naïve patients are yet to be elucidated.

We present a case of a patient suffering from systemic mastocytosis in which rocuronium followed by sugammadex was used and serum tryptase levels were monitored during the perioperative period. We also performed a systematic review of the published literature on the use of sugammadex in patients with systemic mastocytosis.

## Case report

### Patient information, clinical findings and diagnose

The patient consented to publication and signed the informed consent. We present the case of a 50-year-old woman (184 cm, 66 kg) with American Society of Anesthesiologists (ASA) physical status II, diagnosed with systemic mastocytosis with c‑kit D816 mutation and expression of CD2 and CD25 with cutaneous symptoms (urticaria) and episodes of systemic anaphylaxis. She had been treated with subcutaneous epinephrine at that time. The patient had a personal history of Hashimoto’s thyroiditis and drug-induced hepatitis. In addition, she had previously undergone left parathyroidectomy, subsequently suffering from generalized urticaria associated with nonsteroidal anti-inflammatory drugs (NSAID) in the early postoperative period. The patient had no other medical history. She was scheduled for right hemithyroidectomy to solve a follicular neoplasia of the right thyroid lobe. She did not present predictors of difficult airway management. Her daily medication included sodium cromoglycate 100 mg/4 h and ebastine 20 mg/24 h. Physical examination revealed erythematous lesions on the trunk and lower limbs. The baseline serum tryptase determination was 140 ng · ml^−1^. The tryptase determination technique was fluoroenzyme immunoassay (immunoCap 250® [Phadia, Uppsala, Sweden]), reference values: 0–11 ng · ml^−1^.

### Perioperative management

The patient was evaluated by the allergology department prior to the surgical intervention in order to carry out a multidisciplinary approach to the most appropriate drugs for perioperative management. In this assessment, it was concluded that the only NSAIDs that could be used were paracetamol and celecoxib. In addition, the pertinent recommendations were made in case the patient had to undergo general anesthesia. Thus, she was premedicated with oral prednisone 0.5 mg · kg^−1^ 1h prior to the surgery, and with intravenous dexchlorpheniramine 5 mg, ranitidine 100 mg, and montelukast 10 mg immediately before anesthetic induction. In addition, midazolam 3 mg was administered in the preanesthesia room. Intraoperative monitoring included electrocardiogram, heart rate (HR), bispectral index (BIS), pulse oximetry (SpO_2_), noninvasive blood pressure (NIBP) and train-of-four (TOF). Vital signs at the arrival to the operating room were: NIBP 132/82 mm Hg, HR 90 bpm, SpO_2_ 100%. Anesthetic induction was performed with etomidate 0.2 mg · kg^−1^, remifentanil in continuous perfusion 0.1 mcg · kg^−1^ · min^−1^ and rocuronium 0.6 mg · kg^−1^. Intraoperative maintenance was performed using balanced anesthesia, with desflurane 0.7–0.9 MAC (minimal alveolar concentration) and remifentanil 0.05–0.1 µg · kg^−1^ · min^−1^. The patient showed hemodynamic stability, adequate peripheral saturation (SpO_2_ 98–100%) and adequate neuromuscular relaxation (TOF 0–1/4) throughout the intraoperative period (length of surgery: 115 min). At the end of the procedure, neuromuscular blockade was reversed with sugammadex 2 mg · kg^−1^ according to TOF response (TOF 3/4) and the patient was transferred to the intensive care unit (ICU) for postoperative monitoring. Her vital signs at the arrival to the ICU were: NIBP 119/74 mm Hg, HR 72 bpm, SpO_2_ 100%. No cutaneous or systemic signs of mast cell release were noticed perioperatively. Postoperative analgesic management was controlled using intravenous paracetamol 1 g/6 h and oral celecoxib 200 mg/24 h.

### Monitoring and results

Blood samples were taken for the determination of serum tryptase in the immediate postoperative period (105 ng · ml^−1^), and at 2 h (103 ng · ml^−1^), at 6 h (104 ng · ml^−1^) and 24 h (105 ng · ml^−1^) postoperatively. She did not present symptoms associated with her underlying pathology, or postoperative complications, and she was discharged 48 h after surgery.

## Methods: systematic review

### Eligibility criteria

All studies published up to January 2021 concerning anesthetic management in systemic mastocytosis were included for this systematic review. It was conducted in accordance with current guidelines on systematic literature reviews, and prospectively registered at International prospective register of systematic reviews (PROSPERO:CRD 42021239209). This manuscript adheres to the applicable Preferred Reporting Items for Systematic Reviews and Meta-Analyses (PRISMA) statement.

### Search strategy

Studies were identified within the electronic databases PubMed and Google Scholar. Search strategy was carried out as follows: in PubMed: systemic mastocytosis[MeSH Terms] OR urticaria pigmentosa[MeSH Terms] AND anesthesia[MeSH Terms], and in Google Scholar: “systemic mastocytosis” OR “urticarial pigmentosa” AND anesthesia OR “general anesthesia” OR “sugammadex” NOT “regional anesthesia”.

### Study selection

All studies published up to January 2021 in English, Spanish, or German were eligible. No type of document restriction was applied, and no methodology filters were used. Titles and abstracts of retrieved papers were screened for relevance. Selection of articles was carried out using predefined screening criteria. Disagreements regarding inclusion were resolved via discussion among the authors.

**Fig. 1:**
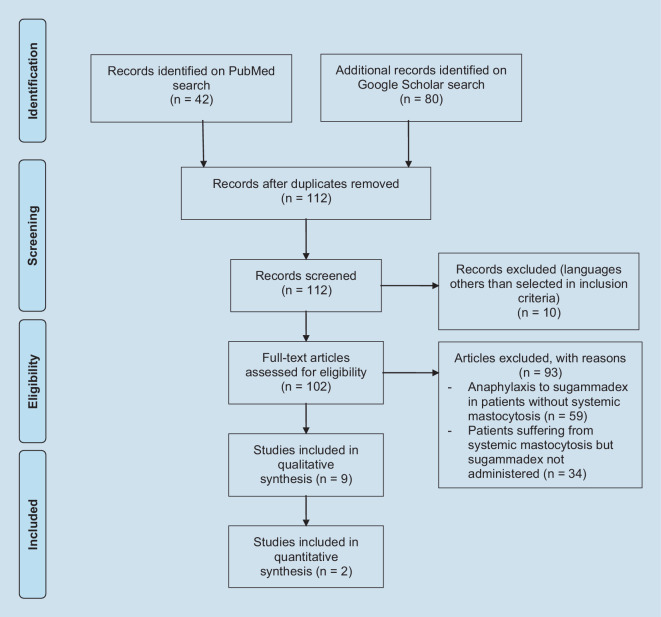
PRISMA flow diagram

The reasons for excluding studies were duplicated papers, publications reporting anaphylaxis to sugammadex in patients not suffering from systemic mastocytosis, or patients suffering from systemic mastocytosis for whom sugammadex had not been administered. A flow-chart illustrating the process of study selection is presented in Fig. 1.

### Data extraction

Using a predefined data extraction form, data were gathered from individual papers. Any discrepancies in the extracted data were resolved via discussion among authors.

Information obtained from reviews were abstracted in a narrative synthesis. Details obtained from case reports were compared and summarized (Table 1). Data regarding the elevation of tryptase levels after sugammadex administration were ordered numerically, as difference between poststimulus and prestimulus values. As no randomized clinical trials were expected to be identified, data extraction was based on conclusions found in reviews and results reported in case reports.

**Table 1:** Reviews and case reports published regarding the use of rocuronium or sugammadex in patients suffering from systemic mastocytosis

Reference	Rocuronium use	Sugammadex use	Tryptase levels monitoring	Observations
Dewachter et al. (2014) [6]	Accepted	Accepted (although there are no data regarding its use)	Not applicable	Review of perioperative management of patients with mastocytosis
Fernández-Candil et al. (2020) [7]	Acceptable alternative	Acceptable alternative	Not applicable	Review of anesthesia in electroconvulsive therapy in special conditions
Unterbuchner et al. (2017) [8]	Used	Used (although no previous data)	Yes, perioperatively	Case report: Rapid sequence induction
De la Fuente Tornero et al. (2017) [9]	Used	Used (in order to reverse a suspected rocuronium anaphylaxis)	Postoperatively, to monitor anaphylaxis	Case report: Patient suffering from Kounis syndrome as onset of systemic mastocytosis
Dewachter et al. (2018) [10]	Used	Used	Postoperatively, to monitor anaphylaxis	Case report: IgE-mediated allergy in a patient with unsuspected mastocytosis
Parra et al. (2020) [11]	Used	Used	Yes, perioperatively	Case report: Scheduled neurosurgery
Bryson et al. (2017) [12]	Used	Not available	No	Case report: electroconvulsive therapy
Aloysi et al. (2016) [13]	Acceptable alternative (not used)	Not available	No	Case report: electroconvulsive therapy
Kumaraswami et al. (2018) [14]	Acceptable (not used)	Acceptable (not used)	No	Case report: vaginal delivery with neuraxial analgesia in mast cell activation syndrome

## Results

Our search yielded 122 articles: 42 in PubMed and 80 in Google scholar. After excluding duplicate articles, 112 articles remained. Exclusions were made for the following reasons: 59 articles describe the appearance of anaphylactic reactions related to the use of sugammadex in patients who did not suffer from systemic mastocytosis, 34 articles describe anesthetic management in patients with systemic mastocytosis without specifying the use of sugammadex, and 10 articles were written in languages other than those included in the inclusion criteria.

### Reviews

Only one review of those published regarding anesthetic management in patients with systemic mastocytosis recommends the administration of sugammadex [6]. Although they found no reason to advise against its use in these patients, they acknowledged that there are no data concerning it.

Moreover, in a review on anesthetic recommendations in electroconvulsive therapy in special conditions [7], the use of full-dose rocuronium reversed with sugammadex in short-term procedures is an acceptable alternative for patients with systemic mastocytosis.

### Case reports

In 4 case reports of patients with systemic mastocytosis the use of sugammadex is reported [8–11]. Two articles justify not using of sugammadex due to its unavailability [12, 13]. In another article, sugammadex is proposed as an acceptable option in pregnant women with mastocytosis, although in the reported case neuraxial anesthesia was used [14].

**Fig. 2:**
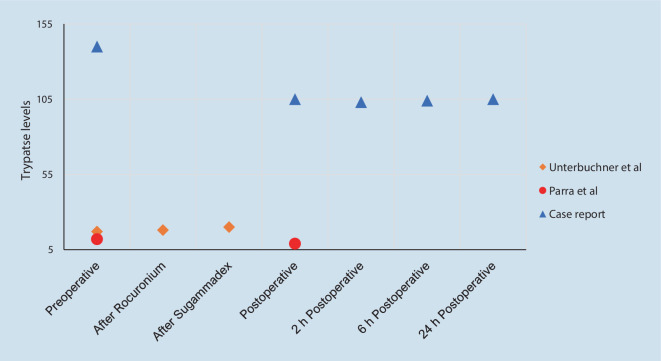
Perioperative evolution of tryptase levels from published case reports that monitored it (Tryptase levels are expressed as ng · ml^-1^) [8, 11]

Only in 2 of the reported cases in which sugammadex was used, was perioperative monitoring of serum tryptases performed. In the article by Unterbuchner et al. the levels of tryptases went from 17 ng · ml^−1^ preoperatively, to 18 ng · ml^−1^ (+5.9%) 30 min after the administration of rocuronium and to 20 ng · ml^−1^ (+11.8% with respect to basal value) 30 min after the administration of sugammadex [8]. In the case reported by Parra et al., the levels of tryptases went from 12 ng · ml^−1^ preoperatively down to 9 ng · ml^−1^ (−25%) in the postoperative period, although it is not specified how much time elapsed from the administration of sugammadex to tryptase determination [11]. Figure 2 shows the evolution of the tryptases levels in the two cases referred to, together with the evolution in the case reported in this article.

## Discussion

To our knowledge, this is the first reported case of monitoring the evolution of serum tryptase levels in a patient with systemic mastocytosis. It was carried out during the first 24 postoperative hours after the administration of rocuronium-sugammadex, showing the absence of mast cell degranulation from clinical and analytical points of view.

Systemic mastocytosis is a hematological disorder characterized by aberrant mast cell accumulation that typically affects the skin and/or the bone marrow. Patients suffering from this pathology may present systemic symptoms secondary to mast cell degranulation. When mast cells are activated, large amounts of mediators are released, causing an anaphylactoid reaction, affecting up to 50% of the patients throughout their lives [15]. Therefore, conventional allergy tests or IgE measurements are not useful for the diagnosis of mast cell degranulation since it is not mediated by IgE [16]. Also, there is no evidence of a higher prevalence of IgE-mediated hypersensitivity in these patients compared to the general population [6]. The measurement of serum tryptase is a simple test with an adequate reproducibility, useful in the diagnosis of all forms of reactions that occur as a consequence of mast cell activation [17, 18].

The incidence of hypersensitivity related to anesthesia and/or surgery is unknown in these patients [6]. During the perioperative period, a large number of stimuli are produced that can provoke mast cell degranulation, such as emotional stress, trauma, alterations in the patient’s temperature, or the administration of histamine-releasing drugs such as morphine. Therefore, the perioperative phase is considered as a potential risk period for these patients. Because multiple and varied stimuli can trigger symptoms, prevention is considered essential. Given the low incidence of the disease, it is not possible to design clinical trials studying the use of drugs in this group of patients. Thus, most of data collected on the safety of anesthetics in these patients are based on case reports; however, there are controversies among some published clinical cases. It must also be taken into account that some guidelines may arbitrarily contraindicate drugs that could be used in certain circumstances since they are based on expert opinions [19].

It is necessary to establish an action plan during the preoperative period by investigating previous events that triggered clinical symptoms in the patient [6]. In the case presented, a consensual action plan was established, admitting the patient 24 h before the procedure and starting premedication with corticosteroids, H1 and H2 antihistamines, and leukotriene receptor antagonists. Furthermore, histamine-releasing drugs including neuromuscular blocking agents such as benzylisoquinolines, or morphine derivatives were avoided. If the information obtained in preclinical studies is applied, it seems logical that the administration of histamine-releasing drugs should be avoided in these patients [8].

Nondepolarizing neuromuscular blockers are the agents most frequently implicated in anaphylactic and anaphylactoid reactions during anesthetic procedures, and rocuronium is the most common [20, 21]; however, conflicting information has been published regarding the possible use of rocuronium in patients suffering from systemic mastocytosis. Rocuronium causes mast cell degranulation by activating a mast cell-specific receptor, the human MRGPRX2. This reaction occurs even in patients without sensitization to this agent, provoking pseudoallergic (nonanaphylactic) reactions [5]. Thus, some authors recommend avoiding it [16, 22–27]; however, other authors recommend using it as an alternative to others that release even more histamine, such as atracurium or succinylcholine [6, 12, 28–32]. The administration of sugammadex has been shown to prevent rocuronium from interacting with the immune system, but anaphylactic reactions induced by rocuronium cannot be completely abolished by sugammadex [33].

The more recent incorporation of sugammadex into the therapeutic arsenal means that there is little information on its appropriate use in these types of patients. Despite this, it is recommended in one of the publications included in this systematic review [6]. The increasing number of case reports and case series in which sugammadex has been shown to be safe in patients with systemic mastocytosis [8–11] supports this recommendation; however, there are few cases in which tryptase levels were monitored to confirm the apparent clinical safety. In the case published by Unterbrucher et al., the levels of tryptases did not change significantly within a small range in comparison to preoperative baseline [8], while in the one published by Parra et al. they even decreased [11]. In our case, tryptase levels fell from the baseline value of 140 to 105 ng · ml^−1^ (−25%). The stimulation of mast cells by the sugammadex-rocuronium complex might be missed, because sugammadex was administered at the end of the surgery and not just after the administration of rocuronium. Quantification of the plasma level of rocuronium at the time of sugammadex administration would have been the gold standard; however, the case report is intended to describe the safety of sugammadex in patients with systemic mastocytosis in the routine clinical practice. Moreover, it is important to note that our patient’s baseline tryptase values were higher than in the other reported cases. Perhaps the maintenance (or even the decrease) of the tryptase values was the consequence of the premedication established [26], masking the possible mast cell degranulation that these drugs could potentially cause. Taking into account that preoperative premedication is not standardized in these patients, we consider it essential to monitor serum tryptase levels throughout the perioperative period.

The clinical case presented, and the literature included in this systematic review confirm that the use of rocuronium followed by sugammadex is safe in patients suffering from systemic mastocytosis; however, it is necessary to have an established perioperative action plan, a consensual premedication, and to avoid other histamine-releasing triggers in these patients.
